# A comprehensive model for heat-induced radio-sensitisation

**DOI:** 10.1080/02656736.2017.1341059

**Published:** 2017-07-05

**Authors:** Sarah Catharina Brüningk, Jannat Ijaz, Ian Rivens, Simeon Nill, Gail ter Haar, Uwe Oelfke

**Affiliations:** Joint Department of Physics, Institute of Cancer Research, Royal Marsden NHSF Trust, Sutton, UK

**Keywords:** Linear quadratic model, thermo-radio-sensitisation, thermal dose, radiation, focused ultrasound

## Abstract

Combined radiotherapy (RT) and hyperthermia (HT) treatments may improve treatment outcome by heat induced radio-sensitisation. We propose an empirical cell survival model (AlphaR model) to describe this multimodality therapy. The model is motivated by the observation that heat induced radio-sensitisation may be explained by a reduction in the DNA damage repair capacity of heated cells. We assume that this repair is only possible up to a threshold level above which survival will decrease exponentially with dose. Experimental cell survival data from two cell lines (HCT116, Cal27) were considered along with that taken from the literature (baby hamster kidney [BHK] and Chinese hamster ovary cells [CHO]) for HT and combined RT-HT. The AlphaR model was used to study the dependence of clonogenic survival on treatment temperature, and thermal dose R^2^ ≥ 0.95 for all fits). For HT survival curves (0–80 CEM43 at 43.5–57 °C), the number of free fit AlphaR model parameters could be reduced to two. Both parameters increased exponentially with temperature. We derived the relative biological effectiveness (RBE) or HT treatments at different temperatures, to provide an alternative description of thermal dose, based on our AlphaR model. For combined RT-HT, our analysis is restricted to the linear quadratic arm of the model. We show that, for the range used (20–80 CEM43, 0–12 Gy), thermal dose is a valid indicator of heat induced radio-sensitisation, and that the model parameters can be described as a function thereof. Overall, the proposed model provides a flexible framework for describing cell survival curves, and may contribute to better quantification of heat induced radio-sensitisation, and thermal dose in general.

## Introduction

The efficacy of radiotherapy (RT) is limited by the relative radio-sensitivities of the tumour and normal tissue surrounding it. Hypoxic cells are generally more resistant to killing by ionising radiation than are normoxic ones. Therefore, when dose escalation is limited by normal tissue toxicity, combining RT with other treatments in order to sensitise these hypoxic targets may be advantageous. Hyperthermia (HT), i.e. mild, sustained heating, is considered to be an effective radio sensitiser [1–5]. Therapeutic ultrasound (ThUS) is a minimally invasive, non-ionising technique for local tissue heating [6,7]. With this technique it may be possible to deliver HT treatments at higher temperatures, but on a shorter timescale than is currently used clinically [8]. The synergistic effects of heat and radiation together with the localised, non-invasive treatment capability of ThUS, mean that its combination with RT offers an exciting potential treatment approach.

In order to enable treatment planning for such combined therapies, however, the underlying biological effects must be quantified, and models put in place to predict them. For such models, describing the cellular effects, in terms of survival curves, are the first requirement. There is ongoing discussion about the underlying biological mechanisms which lead to synergistic heat and radiation induced cell killing. Un- or wrongly-repaired DNA double strand breaks (DSBs) are considered to be the major cause of radiation induced mitotic catastrophe, eventually resulting in cell death. One possible explanation of the enhancement of radiation cell killing by HT may be the inhibition of important DNA repair mechanisms in cells treated [9–13].

## Background

Several mathematical models which describe the observed cellular response to RT (see reviews e.g. [14,15]), or HT ([16–18]) have been proposed. Of these, the linear-quadratic (LQ) model [19] and the thermal dose concept [20] are the most common for RT, HT and ThUS treatment planning.

The empirical LQ-model describes the fraction *S* of cells surviving irradiation with a single fraction of dose *d*, using an exponential of a second-order polynomial characterised by two radio-sensitivity parameters, *α* and *β*.

(1) $$S_{LQ}=e^{-(\alpha d+\beta d^{2})}=e^{-Y}$$

In the original publication, the linear contribution *e*^−α*d*^ was attributed to DSBs resulting from single-track events, whereas the quadratic component *e*^−^*^βd^*2*^^* was introduced to account for DSBs caused by two-track events. Although the LQ-model provides a good fit to experimental data in an intermediate dose regime (∼2 − 10 Gy), both higher and lower doses are less accurately described. Several authors (e.g. [21–26]) have contributed to the discussion on modelling low dose hypersensitivity, or on cell survival in the high dose range and pro- posed adaptations to the original LQ-model formula to overcome this limitation.

The negative exponent *Y = αd + βd*^2^ in Equation (1), known as the “biological effect”, is a dimensionless quantity which can be used to compare the biological response to different treatment modalities. The isoeffective ratio of doses *D_2_* and *D_1_* which yield the same bio- logical effect for a reference modality, and the treatment modality of interest, is defined as “relative biological effectiveness” (RBE).

(2) $$RBE=\frac{D_{2}}{D_{1}}{|}_{Y=const}$$

According to this definition, the RBE of two treatments is a function of the biological effect *Y* and not constant over the whole range of the survival curve. The RBE-weighted dose, *RBE · D_1_*, corresponds to the equivalent dose in units of *D*_2_ needed to obtain the same biological effect *Y* as with treatment at dose *D*_2_.

Although the LQ model has been applied to fit cell survival curves of treatments, such as RT [19] and HT [27,28], with adapted parameters, it was designed for one specific treatment modality, and may therefore not be suitable for describing multi-modality therapies. In particular, it is not suitable for describing HT cell survival curves which give surviving fractions as a function of treatment time at a specific temperature. Treatment time thus represents the “dose parameter” for HT treatments. These curves are characterised by a strong shoulder followed by an exponentially linear decrease of survival with heating time t at a characteristic slope *D*_0_.

(3) $$\frac{\partial Y}{\partial t}=D_{0}$$

The LQ-model is unable to describe the shoulder region of these curves, and overestimates the effect at high (thermal) doses where the quadratic component of the formula dominates. Therefore, HT survival curves have been described by the Arrhenius model [29]. This is based on the kinetics of chemical reactions as described by Arrhenius equations which describe irreversible processes, expressing the rate constant *k* of the reaction as an exponential function of the ratio of the activation energy *E_a_*, and the thermal energy (the product of temperature *T* and universal gas constant *Ʀ*) [20,30–32].

(4) $$k=Ae^{-\frac{E_{a}}{ƦT}}$$

A is a characteristic of the system studied. For HT survival curves, the rate constant *k* (in min^−1^) corresponds to the inverse final slope of the curve, *D*_0_^−1^ [29]. The Arrhenius model therefore provides a description of HT cell survival only in the exponentially linear decay region of the survival curve, the initial shoulder of the curve is not covered by this model description.

In order to allow calculation of biologically equivalent times for HT treatments at different temperatures, and to sum up the effects of inhomogeneous heating profiles, the thermal dose concept [20] was introduced. This is a two case model which expresses heating times *t_X_* at a temperature *T_X_* in terms of “thermal dose” (or CEM43), i.e. equivalent heating time at 43 °C, *t*_43_. “Thermal dose” therefore represents a concept of calculating an RBE-weighted treatment time for HT treatments.

(5) t43=tX·R43 °C-TX with R= 0.25 TX≤43 °C0.5 TX>43 °C

The parameter *R* in Equation (5) depends on temperature and the cell line specific activation energy of the underlying chemical reaction as described by Arrhenius equations. Despite these cell line and temperature dependencies, constant values of 0.5 and 0.25 are commonly assumed for *R* for temperatures ranging from 41 to 45 °C as indicated in Equation (5). Importantly, due to the exponential relation of thermal dose, for high temperatures, deviations in *R* from an approximation of a constant value of 0.5 may significantly influence the calculated thermal dose values and deviate from actual thermal damage [33]. A constant parameter *R* also means that RBE would be independent of the underlying biological effect in this case. Although separate models exist for both RT (LQ-model), and HT (Arrhenius model) cell survival curves, to date no unifying mathematical model description has been proposed. We introduce a modified LQ-model which is able to both describe RT and HT cell survival curves and combinations thereof. The model is validated using a set of previously published cell survival curves, as well as our own data set for single and combination treatments for a temperature range of interest to ThUS mediated HT (45–48 °C).

## Methods

### The AlphaR model

#### Model formulation

Heat-induced radio-sensitisation is believed to be mainly due to an inhibition of DNA repair mechanisms. Thus, for combined RT-HT treatments, a model that reflects this hypothesis is of interest. In the proposed model, we consider the opposing actions of induced damage and its repair. If there were no cellular repair mechanisms, cell survival plotted on a log scale would decrease linearly as a function of treatment “dose” per fraction *D*, i.e. radiation dose or heating time, with a characteristic slope *α*_0_. The underlying damage mechanism could be of an arbitrary origin and might actually be a combination of DNA and other cellular damage, e.g. to the membrane or cellular proteins. Importantly, cells will counteract this damage using a number of repair mechanisms which in turn will be treatment dependent, and are here described by a function F_R_ (*D*). The slope of the cell survival curve (on a log scale), ∂*Y*, is therefore the sum of damage and repair contributions:

(6) $$\frac{dY}{dD}=\alpha_{0}-F_{R}(D)$$

Mathematically, the simplistic approximation for describing the repair function is the approximation with a Taylor series up to linear order:

(7) FRD=αR-2βD D≤DT =αR2β0 D>DT

Here, *α_R_* represents the rate of damage compensation, which is counteracted by a dose-dependent term *2βd*, i.e. damage to the repair potential. There is therefore a limiting dose, *D_T _= α_R_*/(2*βd*), up to which repair mechanisms are active (FR (*D*) > 0). For treatments with doses exceeding *D_T_*, F_R_ (*D*) is 0 (negative values of F_R_ are not meaningful).

Integrating the slope of the survival curve given in Equation (6) and using the repair function F_R_ (*D*) as defined in Equation (7) results in an expression of the biological effect, *Y*, as a function of dose described by a two case model:

(8) YD=∫0DdYdD′dD′=(α0-αR)D+βD2 D≤DT α0D-αR24β D>DT

Below the threshold dose, *D_T_*, the survival curve has a linear quadratic behaviour with parameters *α = α*_0_* − α_R_* and *β*. At doses exceeding *D_T_*, no further repair is possible and cell survival is described by a single exponential.

### Experimental procedure

#### Cell lines and culture conditions

The human cancer cell lines, HCT116 (colorectal carcinoma), and Cal27 (squamous cell carcinoma) were used to generate cell survival curves for testing the model presented. HCT116 cells were cultured in McCoy’s 5 A medium (Gibco, Paisley, UK) containing 1% antibiotics (50 U/ml each of penicillin, streptomycin B and Amphotericin B (Sigma, Poole, UK)), Cal27 cells were grown in DMEM (Gibco, Paisley, UK). All media were supplemented with 10% fetal bovine serum (PAN Biotech, Wimborne, UK). Cells were grown in T80 culture flasks in a humidified atmosphere with 5% CO2 at 37 °C and passaged twice a week using Accutase (Gibco, Paisley, UK). For experiments, cells in exponential growth phase between passages 5 and 20 were used. Regular screening for mycoplasma and bacterial contamination was performed.

#### Cell treatments

Cells were detached from the flask, counted and concentrated to give a cell suspension of 5·10^6^ cells per ml in complete growth medium. 60 μl volumes of cell suspension were transferred to 150 μl thin walled polymerase chain reaction (PCR) tubes (VWR, Lutterworth, UK). For irradiation, sealed tubes were placed in customised blocks of solid water in a small water bath at room temperature. This allowed for reproducible localisation of the cells 1 cm deep in (solid) water in the centre of the open field of a small animal irradiation research platform (SARRP, X-Strahl, Camberly, UK). The machine was operated at 220 kVp, and a 1 mm copper filter was used to harden the beam. The dose rate used was 63.5 mGy/s at the PCR tube location. Monte Carlo simulations and absolute dose measurements were performed to verify the intended dose delivery.

For thermal exposure, cell containing tubes were placed in the central wells of a Biorad Tetrad2 DNA Engine PCR thermal cycler (Hercules, CA), and underwent a three step heating protocol as described in [34]. Plateau heating temperatures and durations ranged from 45 to 48 °C, and 1–80 min, respectively. Heating profiles were verified as described in [34]. A minimum heating time of one minute was chosen, in order to minimise the contribution of thermal effects from heating and cooling gradients to the total thermal dose received by the sample.

For combination treatments, cells were irradiated first, since our pilot control experiments confirmed that the radio-sensitising effect was more pronounced in this order for the cell line used (data not shown). Cells were placed on ice before, after, and between, treatments to minimise cellular activity. Time between treatments was recorded and kept to a maximum of 20 min. It was confirmed that there was no significant difference in clonogenic cell survival for time intervals up to 35 min between irradiation and heat application.

#### Clonogenic assay

After treatment, the cells were plated in triplicates in 6-well plates, or 9 cm Petri dishes, for colony forming assays. Seeding densities were adjusted to yield a final number of approximately 50 colonies per well. These ranged from 50 to 5·10^5^ cells/well. To prevent variations in plating efficiency due to different seeding densities in the wells, differences in seeding densities were accounted for by the addition of irradiated (48 Gy) feeder cells [35] of the same cell line to yield a final cell seeding density of 10^4^ cells/cm^2^. Feeder cells were allowed to attach overnight before treated cells were added. Control plates containing only irradiated feeder cells were plated for each experiment to ensure that no colonies arose from these. Plates were incubated for 9–12 d to allow colony formation.

After macroscopically visible colonies had formed, the culture medium was removed, and colonies were gently rinsed with PBS before being fixed using ice cold 100% methanol. Dried plates were stained with 0.5% Gentian Violet solution in water (Sigma-Aldrich, Schnelldorf, Germany), and washed with tap water to remove unbound stain. Colonies in dried plates were scored if they exceeded a threshold number of 50 cells. Wells with overlapping colonies were excluded from the analysis.

To obtain cell surviving fractions, colony numbers were normalised to the number of cells seeded, as well as to the plating efficiency of the untreated controls, or, in the case of combination treatments, the number of colonies arising when cells were solely thermally exposed. Results from at least three independent repeats were averaged for each data point. Besides the data sets obtained experimentally for HCT116, and Cal27 cells, cell survival data for baby hamster kidney (BHK) [36] and Chinese hamster ovary cells (CHO) [37] cells from the literature were analysed. Table 1 gives an overview of the cell survival data used, and the corresponding treatments.

**Table 1.** Summary of the 42 cell survival data sets used in this study. Indicated are the cell line, treatment duration t at temperature *T*, and radiation dose range *D*. For all combination treatments of RT and HT, RT was delivered first.

HCT116			Cal27			BHK [36]			CHO [37]		
*t* [min]	T [°C]	D [Gy]	*t* [min]	T [°C]	D [Gy]	*t* [min]	T [°C]	D [Gy]	*t* [min]	T [°C]	D [Gy]
0–56	45	–	0–80	45	–	180	43.5	–	20,30,40,60,80	43	0–8
0–28	46	–	0–40	46	–	50	46	–	20	44	0–8
0–14	47	–	0–20	47	–	1	50	–	10,15,20	45	0–8
0–7	48	–	0–10	48	–	0.1	54	–	6,9	46	0–8
1.25	48	0–6	1	48	0–6	0.01	57	–			
1.25,2.5,3.75,5	47	0–6	1,2,3,5	47	0–6						
2.5,5,7.5,10	46	0–6	4	46	0–6						
5,10	45	0–6	8	45	0–6						

#### Data analysis and model fitting

All cell survival data shown are mean values, with standard deviations indicated as error bars (if exceeding the symbol size). In the case of CHO cells, the symbol size of most of the published survival data exceeded the size of potential error bars, which were quoted as standard errors of the mean [37]. Thus, no error bars could be provided for these data sets. Cell survival data sets were fitted to the AlphaR, and the LQ-model using a nonlinear least squared fit in MATLAB (MathWorks^®^, Natick, MA). Fits were performed on averaged data weighted using the uncertainties of each data point ($\left(weighting\;factor\;w={\left(\frac{mean(S)}{std(S)}\right)}^{2}\right)$). The fit parameters obtained were further analysed for their time and temperature dependence using the same fitting approach, and are always given with 95% confidence bounds.

## Results

### Hyperthermia treatments

#### Comparison of the AlphaR and the LQ-model

**Figure 1.**
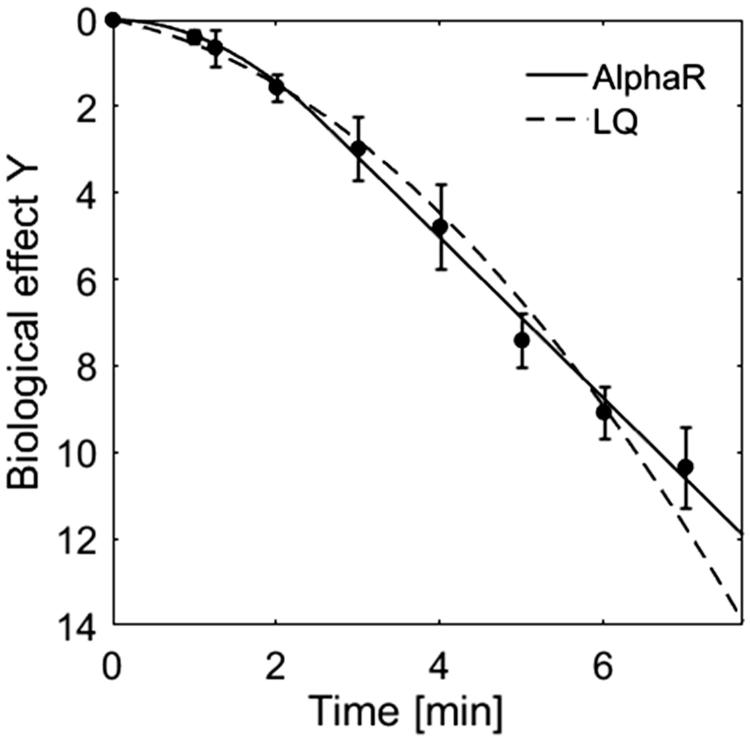
Comparison of the fit of HT (48 °C) cell survival data of HCT116 cells by the AlphaR (solid line) and LQ (dashed line) model. In contrast to the LQ model, the AlphaR model follows the initial shoulder and exponentially linear decay of the data. The corresponding coefficients of determination (R^2^_AlphaR_ = 0.99, R^2^_LQ_ = 0.98) further support this observation.

HT cell survival curves are characterised by an exponentially linear descent following an initial shoulder region. When comparing the fits of the LQ and the AlphaR model (selected results shown in Figure 1) it is clear that the AlphaR model provides a good fit, in both the shoulder and exponential decay regions of the curve, whereas the LQ model cannot describe such a behaviour and overestimates cell killing at high thermal doses.

For all cell lines studied, in the case of HT treatments *α_R_* equals *α*_0_. The number of free model parameters therefore reduces to two in this case, namely *α*_0_ and *β*. Although this is the same number of parameters as used by the LQ-model, the AlphaR model provides the better fit in terms of coefficients of determination (R^2^_AlphaR _= 0.99, R^2^_LQ_ = 0.98). This was expected as the LQ-model represents a sub case of the AlphaR model and fits will therefore always be inferior, or equal to the AlphaR model.

#### Comparing treatments at different temperature

**Figure 2.**
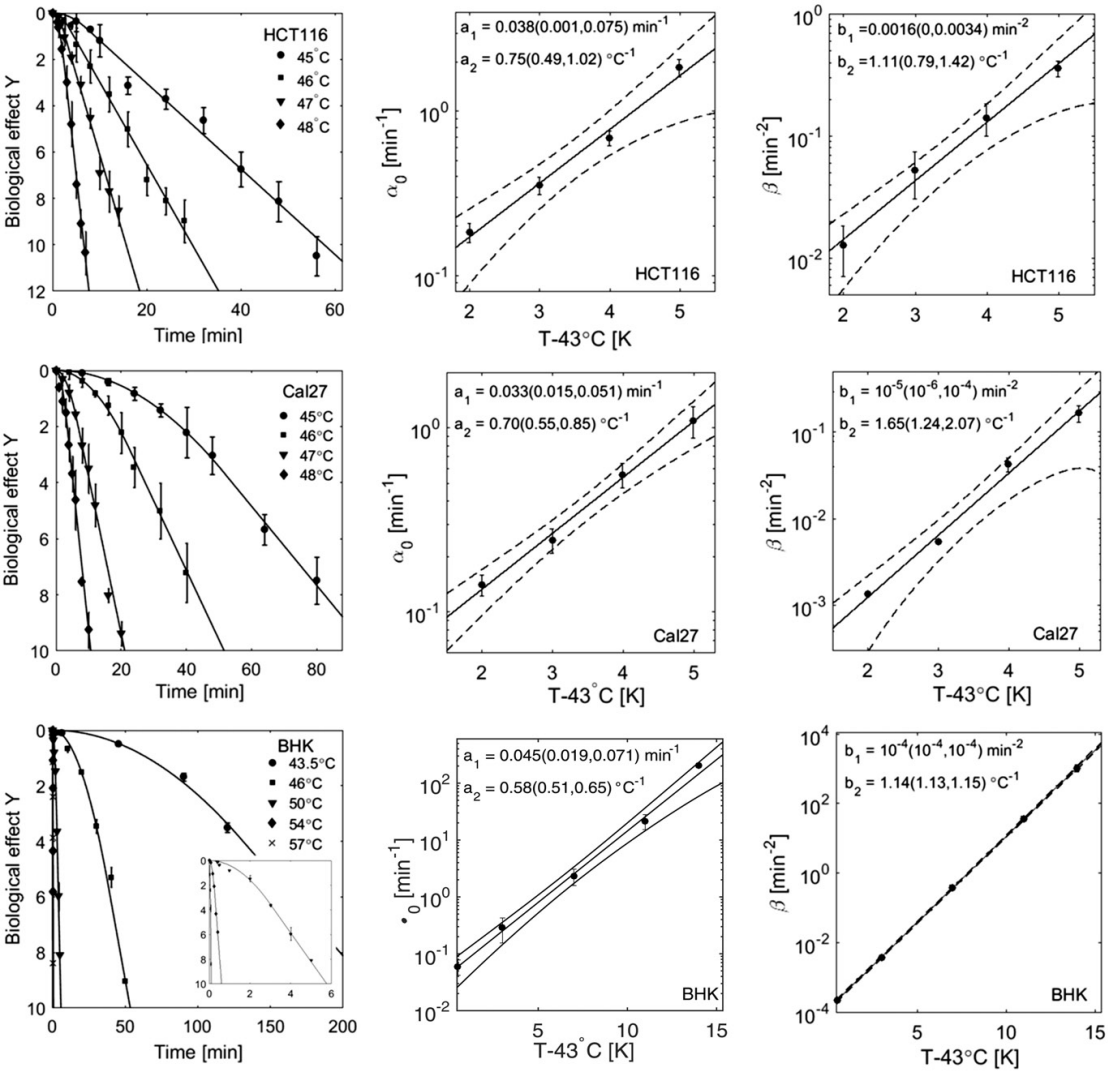
HT cell survival curves at various treatment temperatures of three different cell lines (top: HCT116, middle: Cal27, bottom: BHK [36]) fit by the AlphaR model. The corresponding fit parameters, *α*_0_ and *β*, are plotted as a function of the difference of the heating temperature and 43 °C. This dependence is well described by an exponential fit (solid line, 95% confidence bounds are shown as dashed lines) with the parameters of this exponential (*a*_1_ and *a*_2_, or *b*_1_ and *b*_2_) being shown here with the corresponding 95% confidence bounds.

Figure 2 shows survival curves for HCT116, Cal27 and BHK cells fit by the AlphaR model together with the corresponding temperature dependences of *α_0_* and *β*. The increase of the fit parameters with temperature is well described by an exponential.

$$\alpha_{0}\left(T\right)=\;a_{1}\cdot e^{a_{2}\left(T-43{}^{\circ}C\right)}$$

(9) $$\beta \left(T\right)=\;b_{1}\cdot e^{b_{2}(T-43{}^{\circ}C)}$$

For BHK cells, a very large range of treatment temperatures was covered (43.5 − 57 °C), resulting in tight 95% confidence intervals for the exponential fits.

#### Expressing thermal dose in terms of the AlphaR model

For HT treatments the RBE, see Equation (10) is defined as the isoeffective ratio of treatment times, *t_T_*, at temperature, *T*, and the equivalent time at the reference temperature 43 °C, *t_43_*. Using the AlphaR model, and assuming the threshold doses *D_T_* (43 °C) of heating at 43 °C to be lower than the threshold at higher temperatures *D_T_* (*T*), this ratio is expressed as:

(10) $$RBE\left(T\right)=\frac{t_{T}}{t_{43}}{|}_{Y\;=\;const}$$

For a given biological effect *Y*Equation (8) can be solved for the treatment time (indicated as ‘*D*’ in Equation (8)) providing a description of RBE as a function of the biological effect using the AlphaR model parameters if this expression is inserted in the above Equation (10).

In their thermal dose concept, Sapareto and Dewey assumed a constant RBE over the whole range of the survival curve. The AlphaR model description of RBE is, however, not independent of the biological effect unless the ratio $\frac{\alpha_{0}^{2}}{\beta }$ was constant for a specific temperature, i.e. independent of the underlying biological effect.

(11) $$if\;\frac{\alpha_{0}^{2}}{\beta }=const\to RBE\left(T\right)=\;\sqrt{\frac{\beta (43\;{}^{\circ}C)}{\beta (T)}}=\frac{\alpha_{0}(43\;{}^{\circ}C)}{\alpha_{0}(T)}$$

For the exponential temperature dependence of *α*_0_ and *β* described in Equation (9), this condition for constant RBE would be fulfilled if the relation between the exponents *b_2 _=2a_2_* is valid.

(12) $$\frac{\alpha_{0}^{2}}{\beta }=\frac{a_{1}^{2}}{b_{1}}e^{\left(2a_{2}-b_{2})(T-43\;{}^{\circ}C\right)}={const\;if\;2a}_{2}=b_{2}\;$$

**Figure 3.**
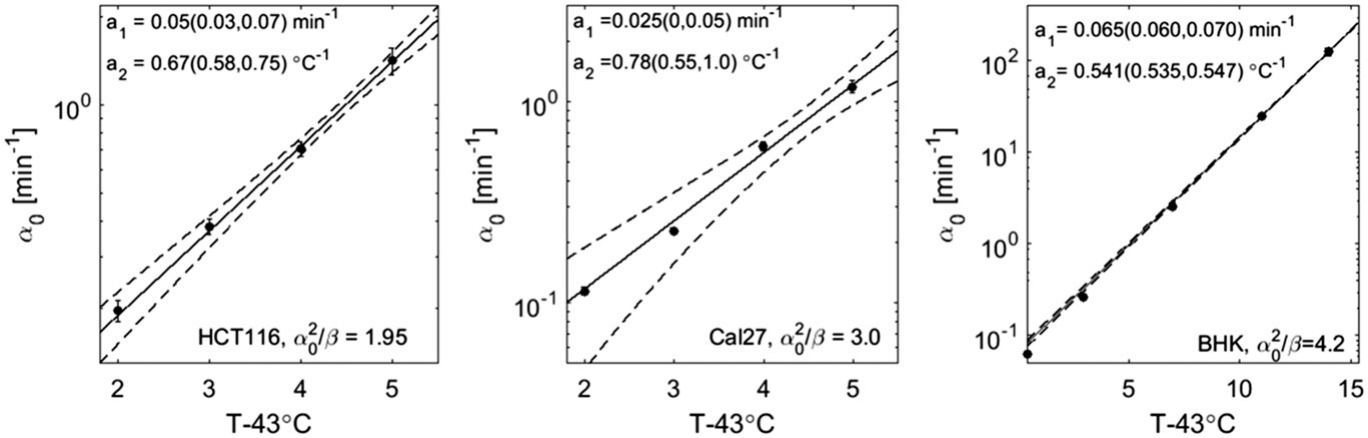
Temperature dependence of *α*_0_ fit to HT cell survival curves of HCT116 (left), Cal27 (middle), and BHK (right) cells under the constraint of a constant ratio $\frac{\alpha_{0}^{2}}{\beta }$ as indicated.

For the cell lines studied, the ratios of *b*_2_ and *2a*_2_ with 95% confidence bounds were 0.74 ± 0.33 (HCT116), 1.18 ± 0.38 (Cal27) and 0.98 ± 0.12 (CHO). Within the range of uncertainties of the fit parameters, RBE may therefore be constant over the whole range of the survival curve. We therefore re-analysed the cell survival data under the constraint of a constant ratio of $\frac{\alpha_{0}^{2}}{\beta }$. For each cell line studied, the parameter ratio which provided the best overall fit to all survival curves at different temperatures in terms of the sum of the coefficients of determination of all survival curves was applied. Under this constraint all survival data could be fitted with coefficients of determination exceeding 0.95, and Figure 3 shows the resulting temperature dependencies of *α*_0_. The thermal dose parameter *R* (see Equation (5)) can now be expressed in terms of the model parameter:

(13) $$\begin{array}{l} \text{R}={\text{e}}^{-\text{a2}}=0.\text{51 }\left(0.\text{48},\;0.\text{54}\right)\text{for HCT116}\\ \text{R}=0.\text{46 }\left(0.\text{54},\;0.\text{38}\right)\text{for Cal27}\\ \text{R}=0.\text{582 }\left(0.\text{58}0,\;0.\text{584}\right)\text{ for BHK} \end{array}$$

### Thermo-radio-sensitisation

#### Influence of the heating protocol

**Figure 4.**
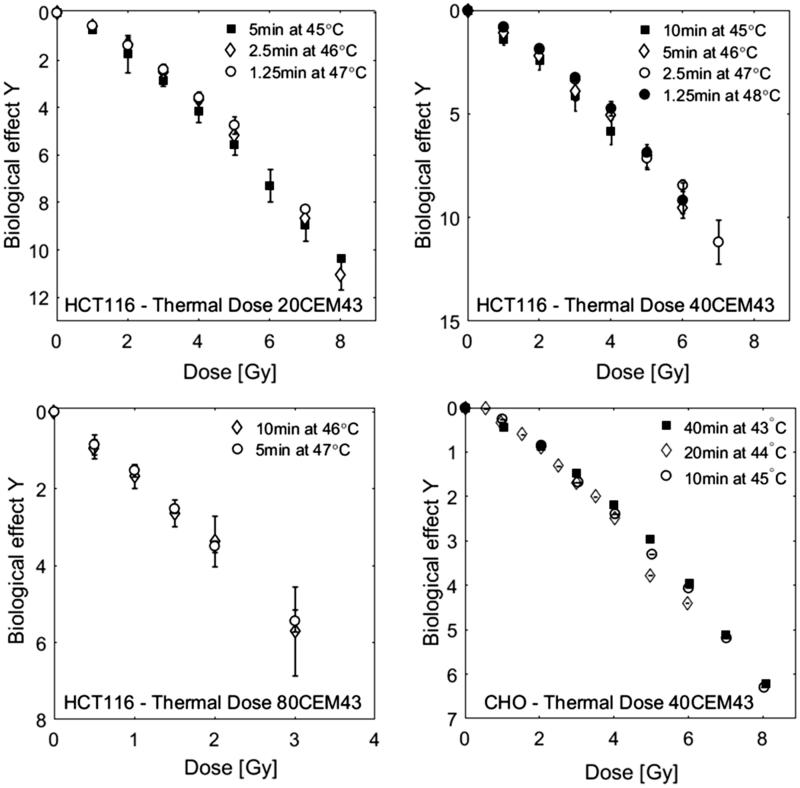
Comparison of HCT116 and CHO [37] cell survival curves for combination treatments of RT and HT for the same thermal dose (20–80 CEM43), but different heating temperatures and durations as indicated in the legends. Cell survival data were normalised to 100% after HT treatment. Within the range of uncertainties of the clonogenic assay, there is good agreement between data sets originating from different time/temperature combinations.

In order to quantify the radio-sensitising effects of different time-temperature combinations applied after irradiation, cell survival curves obtained with a constant thermal dose but different heating temperatures and duration are compared for CHO and HCT116 cells (see Figure 4). There was no statistically significant difference between cell survival curves obtained with a range of heating combinations for thermal doses between the 20–80 equivalent minutes tested for HCT116, and 40 equivalent minutes for CHO cells. The radio- sensitising effect is therefore considered to be the same over the temperature range covered and may be quantified by thermal dose.

#### Thermo-radio-sensitisation modelling with the AlphaR model

**Figure 5.**
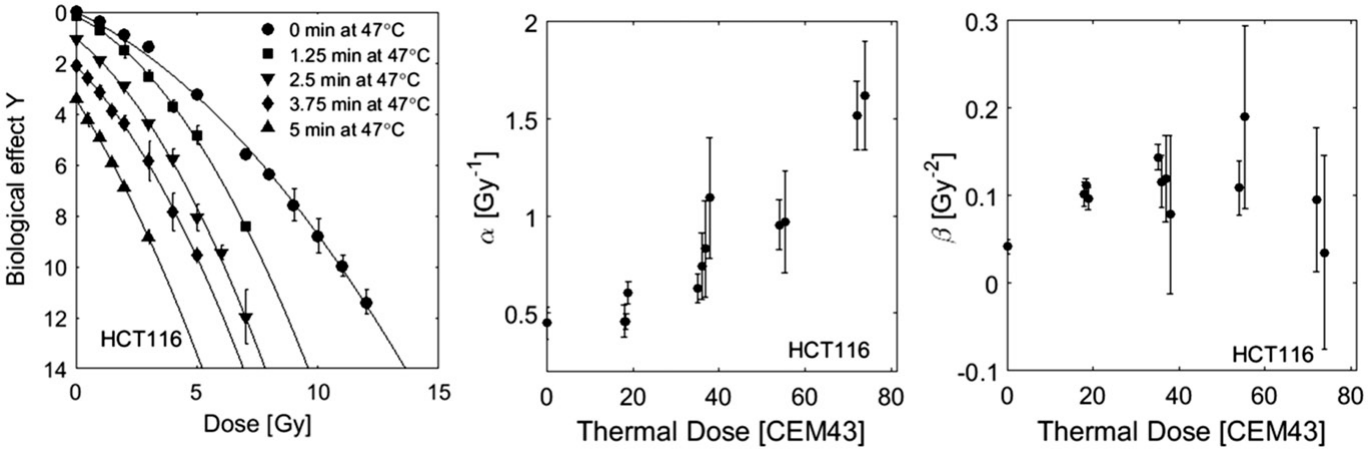
Selected RT-HT cell survival curves of HCT116 cells heated at various times at 47 °C fitted by the LQ part of the AlphaR model (left). For better visualisation of the data, curves are shown with the respective off set in survival after HT treatments. The corresponding fit parameters *α* (middle) and *β* (right) for all survival curves listed in Table 1 are shown as a function of thermal dose. Thermal dose *t_43_* was calculated according to Equation (10) under the assumption of a constant RBE calculated using Equation (11), and the respective values for *α_0_* as indicated in Figure 3.

Examples of survival curves for irradiated and heated HCT116 cells are shown in Figure 5 together with the corresponding fits using the AlphaR model. Due to the experimental limitations that prevented the treatment of more than 10^6^ cells simultaneously, the data range provided does not cover the exponentially linear regime of the cell survival curve. This means that the fit of *α*_0_ and *α_R_* is prone to large uncertainties, making it impossible to draw conclusions about the individual thermal dose dependencies of these parameters. We therefore restrict the discussion to the LQ branch of the AlphaR model (see Equation (8) for doses below the threshold *D_T_*). The thermal dose dependence of *α* (*α = α_0 _− α_R_*) and *β* is analysed for all RT-HT survival curves indicated in Table 1. Thermal dose (*t_43_*) was calculated according to Equation (5) using the values of the thermal dose parameters *R* calculated in Equation (13) for HCT116 and Cal27 cells. Since such data was not available for CHO cells, here a value of 0.5 was used for *R* as suggested in the original thermal dose description.

**Figure 6.**
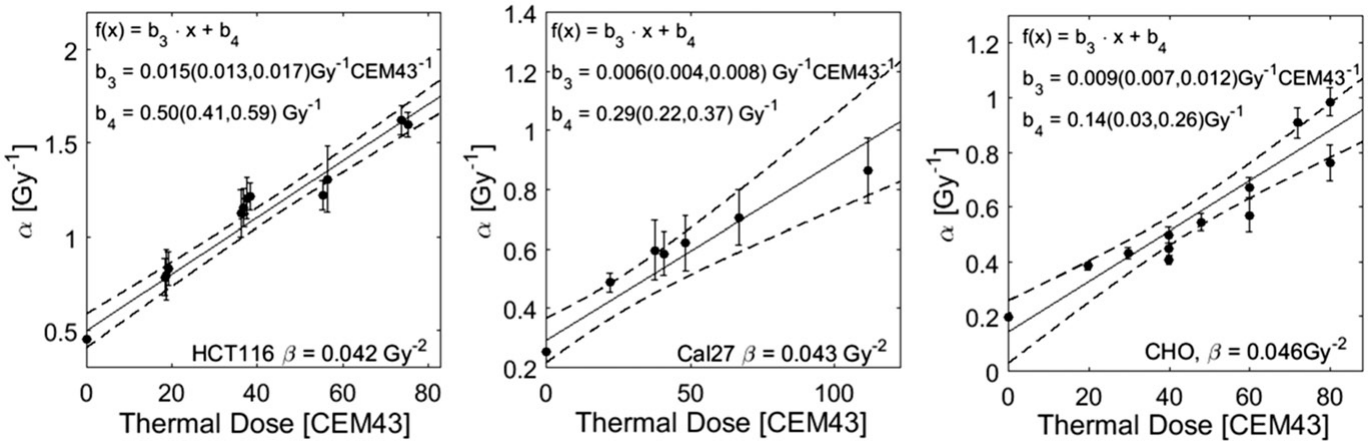
Fitparameter *α* as a function of thermal dose given in addition to RT treatment. Thermal dose was calculated according to Equation (5) using the values of R calculated in Equation (13). The parameter roughly follows a linear increase with thermal dose. The fit was performed under the constraint of a constant value *β* as indicated in this figure. For all data sets the resulting coefficients of determination R^2^ were greater than 0.95 (survival curves are not shown).

Whereas a steady increase of *α* with thermal dose is observed, there is no clear correlation between *β* and thermal dose. We therefore re-fit the data under the assumption of a constant *β* and used the values obtained from a fit of the RT curve without heating (*β*_HCT 116 _= 0.042 Gy^−2^, *β*_Cal27 _= 0.043 Gy^−2^, *β*_CHO _= 0.046 Gy^−2^). The corresponding coefficients of determination were >0.95 for all fits, indicating that the assumption of a constant *β* still provides acceptable fits to the cell survival data. The respective results for *α* are shown in Figure 6 (left) and display a linear increase with thermal dose. The same fitting approach was applied to CHO and Cal27 cell survival data. The thermal dose dependencies of α for these cell lines are shown in Figure 6 (middle and right).

## Discussion

For both treatment modalities, HT and RT, individual model approaches have been proposed in the past and used to describe the respective cell survival curves. While the characteristic continuously bending RT cell survival curves are well described by the linear quadratic model in the intermediate dose regime (≈1 − 10 Gy), the Arrhenius model has frequently been used to describe heat-induced cell toxicity. Although both models work reasonably well for their individual treatment modality, they are not applicable to other treatment modalities.

This motivates the need for a unifying model capable of describing cell survival curves of arbitrary origin. The advantage of the AlphaR model over existing modelling approaches is its flexibility to describe cell survival curves of both of these treatment modalities accurately. Moreover, the AlphaR model overcomes some of the limitations of the individual cell survival models, such as the description of the whole HT survival curve, including its strong shoulder which cannot be modelled by the Arrhenius equation. This model should also improve quantification of heat induced radio-sensitisation, and the understanding and interpretation of thermal dose in general, which are of particular importance for combination therapies as discussed in this article. The AlphaR model provides great flexibility in modelling cell survival curves of various origins. It was motivated by the action and counter action of cellular damage and repair mechanisms. This is of particular interest for an application of the model to combination therapies of HT and RT, since one of the key effects responsible for the synergistic effect of these treatments is a reduction in repair capacity due to the inhibition of important pathways [9,11].

The AlphaR model provides a simple mathematical description that does not necessarily require more fit parameters than the widely used linear quadratic model to provide fits of equal or better quality – here evaluated in terms of coefficients of determination. For the analysis of model parameters as a function of heating duration and/or temperature, it is essential to evaluate several treatment combination data sets originating from different cell lines. In this work clonogenic survival curves for two cell lines (HCT116 and Cal27) were measured for a number of treatment combinations. As a comparison for each treatment modality an additional data set from the literature [36,37] was considered. Although it would be desirable to include even more data in this analysis, this was difficult because of the lack of consistent data in the literature.

For HT treatments, the AlphaR model describes the shape of the survival curve well in both the shoulder and the exponential decay region using only two fit parameters. In this case *α_R_* was found to be equal to *α*_0_. This may imply that below the threshold heating time *D_T_* (see Equation (7)) thermal damage would be reversible if repair were fully functional, whereas the damage is irreversible for treatment times exceeding *D_T_*.

The temperature dependence of *α*_0_ and *β* for HT treatments at different temperatures followed an exponential. The model parameters *α*_0_ and $\sqrt{\beta }$ can therefore be interpreted as rate constants of chemical reactions as described by Arrhenius equations. With this description it was possible to provide a description of the RBE-weighted, i.e. “thermal”, dose as a function of heating time and duration. Due to relatively large uncertainties in the fit parameters obtained, it was not possible to prove that RBE may not be constant over the whole range of the survival curve as assumed by Sapareto’s and Dewey’s thermal dose concept. However, it would not be surprising if there was a change in RBE between the shoulder and exponential decay region of the survival curve due to potential changes in the cellular response pathways activated, or the overcoming of activation energies of protein denaturation. In such a case, the AlphaR model could provide the mathematical tools for calculating RBE at various stages along the survival curve. Further analysis of more cell survival data, in particular of different cell lines is needed to better understand the relation between HT treatments at different temperatures and to accurately summarise complex heating profiles. The data sets used in this analysis were fitted well (i.e. coefficients of determination R^2^ always >0.95) under the assumption of a constant RBE over the whole range of the survival data. This assumption further reduced the number of free model parameters to one (*α*_0_), however, the constant ratio $\frac{\alpha_{0}^{2}}{\beta }$ used was cell line dependent ($\frac{\alpha_{0}^{2}}{\beta }\left(HCT116\right)=1.95,\;\frac{\alpha_{0}^{2}}{\beta }$ (Cal27) = 3.0, $\frac{\alpha_{0}^{2}}{\beta }\;\left(BHK\right)=4.2$), and could only be obtained from the analysis of multiple HT cell survival curves at different temperatures.

For combination treatments, the range of the data provided was not sufficient to determine all parameters of the AlphaR model, since it did not cover the exponentially linear regime of the survival curve needed for the determination of α_0_. Analysis of the data for combination treatments therefore had to be restricted to the LQ branch of the AlphaR model which is applicable for treatment doses below the threshold dose *D_T_* (see Equation (7)). It is important to note, that for combination treatments, survival was normalised to 100% after heat treatment alone. The AlphaR model is able to describe the surviving fraction correctly both after heat treatments alone (i.e. the offset of on the y-axis of the curve) and as a function of additional radiation dose. This is another indication of why the AlphaR model may be preferable over a pure LQ-model. Considering that the synergism between heat and radiation may be due to an inhibition of DNA double strand break repair, it might be a valid assumption to use a constant value for the *α*_0_ parameter independent of the heating time and duration used. In this case, a potential increase of α with thermal dose may be due to a reduction in the repair capacity of the cell, i.e. a reduction of *α_R_*. However, it was not possible to test this hypothesis with the data provided. Limitations in the range of surviving fractions covered were due to the experimental procedure for clonogenic assays and the cell lines used. In order to obtain data points for surviving fractions on the order of 10^−6^, several million cells have to be treated homogeneously (i.e. obtain the same (thermal) dose) and plated in the same dish at the correct seeding density to provide a sufficient number of countable colonies. With the experimental set-up used, this was, however, not feasible due to a limited number of PCR tubes that could be treated simultaneously. Other cell lines may be easier to analyse if they display an earlier transition to the exponentially linear regime of the cell survival curve. The observation that a transition to an exponential linear dose dependence should occur for radiation treatments is described in detail e.g. in [38,39].

Several groups have previously reported on describing the effects of heat induced radio- sensitisation by the LQ-model. However, there is currently ongoing discussion about the heating time and temperature dependence of the LQ model parameters. Consistent cell survival curve data covering a broad range of thermal doses from various time temperature combinations (as e.g. given in [37]) are rare, making it difficult to evaluate dependencies. To date, several approaches have been proposed: Dikomey et al. reported a linear dependence of both *α* and *β* on heating time at a specific temperature, but drew no conclusions about unifying thermal dose dependence. Later, Xu et al. [40,41] reported increasing values of α, but observed no change in *β* for human NSY and HCT15 cells treated with mild HT (41.1 °C, 1 h). This approach was adapted by Myerson et al. for their study of clinical results of patient response modelled with the assumption of a constant *β* parameter [42]. More recently, Franken et al. have reported both increasing and decreasing values of *α* with heating temperature [43]. Modelling studies requiring heating time and temperature dependent LQ parameters for equivalent dose calculations of patient treatment plans for RT-HT combination treatments, have applied a number of model descriptions: Linear and exponential increase of *α* with treatment mean temperature but constant *β* [27]; Piece-wise interpolated parameters [28]; exponential increase of both *α* and *β* with treatment temperature [44]. Except for the study conducted by Dikomey et al. all of these applications focused on mild HT applications with temperatures ranging between 39 and 43 °C. Our data analysis suggests that, for the cell lines and thermal dose range studied (at temperatures exceeding 43 °C), thermal dose may be a good indicator of the degree of radio-sensitisation achieved. But it is indeed difficult to define a specific thermal dose dependence of *α* and *β*, i.e. linear, exponential or no increase with thermal dose. This may be due to the fact that very similar cell survival data sets may lead to different fit results since changes in one parameter may partially be accounted for through the other parameter(s). We therefore decided to minimise the number of free parameters by assuming a constant value for *β*. Despite this simplification, it was still possible to describe the survival data reasonably well (R^2^ always >0.95). The ambiguity of the model fit is particularly clear in the case of CHO cells for which a linear increase of *β* with heating time was originally reported. Therefore, care should be taken when reporting thermal dose dependencies of model parameters, and minimisation of the number of parameters should be considered by applying reasonable simplifications as proposed in this case.

Although this article, and the proposed AlphaR model focus entirely on the cellular response of RT, HT and RT-HT treatments in terms of clonogenic cell survival *in vitro*, it is important to note, that direct cytotoxicity and radio sensitisation at a cellular level are not the only biological effects associated with such treatment combinations. In general, heat-induced cellular effects are expressed as damage to cytoplasmic, membrane and cytoskeletal proteins [9,12,45]. Significant damage to these proteins may induce apoptotic and/or autophagic pathways, whereas uncontrolled coagulation necrosis only occurs in response to temperatures exceeding 50 °C [34]. The conformation of proteins and lipids is affected by the ambient temperature, which in turn influences a protein’s functionality. While lipid aberrations are reversible, they may still affect the rate of protein aggregation, which is considered to be the major cause of heat induced cell death [12,46]. The acute cellular response to heat shock is expressed as an increased synthesis of molecular chaperones (heat shock proteins [Hsp]) that assist in protein folding and can compensate for the increased number of misfolded proteins produced. This enables prolonged survival of the cell at elevated temperatures and may explain the build up of thermo tolerance.

Besides biological effects on a cellular level, a number of physiological effects have been reported for HT treatments *in vivo*. These include changes to the tumour micro-environment (such as pH), enhanced tissue perfusion and vascular changes which may enhance tissue- reoxygenation, as well as the activation of immunological response mechanisms [47–49] (see e.g. [50] for a detailed review). In contrast to the proposed thermal dose concept for cytotoxic effects *in vitro*, which allows for a conversion of treatment times at different temperatures to those at 43 °C, these physiological effects may indeed be dependent on the specific temperature regime, making a comparison of treatments *in vivo* at different temperatures difficult. In particular, alterations in tumour oxygenation may greatly influence the effect of heat-induced radio-sensitisation. Whereas mild HT will enhance tissue perfusion and improve tissue re-oxygenation, resulting in a more effective cell kill upon irradiation, ablative techniques, such as high intensity focussed ultrasound therapy may result in vessel occlusion and oxygen depletion in the tissue. Similarly, the activation of the immune response may already be activated at non-lethal temperatures in the fever range, but different immunomodulatory effects may be present at cytotoxic temperatures (>43 °C) [47].

In order to predict and plan treatment response to combination treatments of HT and RT in patients, it is therefore important to take both cellular and physiological effects into account. Correct modelling of the biological effects on a cellular level *in vitro* may therefore only be considered a first step towards more sophisticated treatment planning.

## Conclusions

The proposed AlphaR model was suitably accurate for describing cell survival curves originating from HT, RT treatments and their combination. The new model overcomes some of the limitations of the LQ model, and provides equivalent or better fits of cell survival curves, which could be quantified in terms of superior coefficients of determination. It was possible to relate the model parameters to existing concepts of thermal dose, and to express them as a function thereof. This formulation therefore holds great potential for future treatment planning applications of focussed ultrasound mediated heating in combination with RT, and may in general be of interest for application to a wide range of combinative treatment modalities.

## References

[1] Horsman M, Overgaard J. (2007). Hyperthermia: a potent enhancer of radiotherapy. J Clin Oncol 19:418–26. 10.1016/j.clon.2007.03.015 17493790

[2] Raaphorst G, Yang D, Ng C. (2000). Comparison of survival and DNA double strand breaks for mild hyperthermia and low dose rate/pulsed low dose rate irradiation in human cells. J Therm Biol 25:305–11. 10745127 10.1016/s0306-4565(99)00103-5

[3] Rao W, Deng ZS, Liu J. (2010). A review of hyperthermia combined with radiotherapy/chemotherapy on malignant tumors. Crit Rev Biomed Eng 38:101–16. 21175406 10.1615/critrevbiomedeng.v38.i1.80

[4] Rozin-Towle L, Pirro J. (1991). The response of human and rodent cells to hyperthermia. Int J Radiat Oncol Biol Phys 20:751–6. 2004951 10.1016/0360-3016(91)90018-y

[5] Kampinga HH. (2006). Cell biological effects of hyperthermia alone or combined with radiation or drugs: a short introduction to newcomers in the field. Int J Hyperthermia 22:191–6. 16754338 10.1080/02656730500532028

[6] ter Haar G, Coussios C. (2007). High intensity focused ultrasound: physical principles and devices. Int J Hyperthermia 23:89–104. 17578335 10.1080/02656730601186138

[7] ter Haar G, Coussios C. (2007). High intensity focused ultrasound: past, present and future. Int J Hyperthermia 23:85–7. 17578334 10.1080/02656730601185924

[8] Mallory M, Gogineni E, Jones GC, *et al* (2016). Therapeutic hyperthermia: the old, the new, and the upcoming. Crit Rev Oncol Hematol 97:56–64. 26315383 10.1016/j.critrevonc.2015.08.003

[9] Lauber K, Brix N, Ernst A, *et al* (2015). Targeting the heat shock response in combination with radiotherapy: sensitizing cancer cells to irradiation-induced cell death and heating up their immunogenicity. Cancer Lett 368:209–29. 25754814 10.1016/j.canlet.2015.02.047

[10] Lauber K, Ernst A, Orth M, *et al* (2012). Dying cell clearance and its impact on the outcome of tumor radiotherapy. Front Oncol 2:1–14. 10.3389/fonc.2012.00116 PMC3438527 22973558

[11] Oei AL, Vriend LEM, Crezee J, *et al* (2015). Effects of hyperthermia on DNA repair pathways: one treatment to inhibit them all. Radiat Oncol 10:165. 26245485 10.1186/s13014-015-0462-0 PMC4554295

[12] Sugahara T, Van Der Zee J, Kampinga HH, *et al* (2008). Kadota Fund International Forum 2004. Application of thermal stress for the improvement of health, 15–18 June 2004, Awaji Yumebutai International Conference Center, Awaji Island, Hyogo, Japan. Final Report. Int J Hyperthermia 24:123–40. 18283589 10.1080/02656730701883675 PMC2765467

[13] Mantso T, Goussetis G, Franco R, *et al* (2016). Effects of hyperther- mia as a mitigation strategy in DNA damage-based cancer therapies. Sem Cancer Biol 37:96–105. 10.1016/j.semcancer.2016.03.004 27025900

[14] Andisheh B, Edgren M, Belki´ c. D, *et al* (2013). A comparative analysis of radiobiological models for cell surviving fractions at high doses. Technol Cancer Res Treat 12:183–92. 23098282 10.7785/tcrt.2012.500306

[15] Besserer J, Schneider U. (2015). A track-event theory of cell survival. Z Med Phys 25:168–75. 25432332 10.1016/j.zemedi.2014.10.001

[16] Wright NT. (2013). Comparison of models of post-hyperthermia cell survival. J Biomech Eng 135:51001. 24231957 10.1115/1.4023981

[17] Feng Y, Oden JT, Rylander MN. (2008). A two-state cell damage model under hyperthermic conditions: theory and in vitro experiments. J Biomech Eng 130:041016. 18601458 10.1115/1.2947320 PMC2869433

[18] Mackey M, Roti Roti JL. (1991). A model of heat-induced clonogenic cell death. J Theor Biol 156:133–46. 10.1016/s0022-5193(05)80669-1 1640720

[19] Fowler JF. (1989). The linear-quadratic formula and progress in fractionated radiotherapy. Br J Radiol 62:679–94. 2670032 10.1259/0007-1285-62-740-679

[20] Sapareto SA, Dewey WC. (1984). Thermal dose determination in cancer therapy. Int J Radiat Oncol Biol Phys 10:787–800. 6547421 10.1016/0360-3016(84)90379-1

[21] Lind BK, Persson LM, Edgren MR, Hedlo I. (2003). Repairable – conditionally repairable damage model based on dual poisson processes. Radiat Res 160:366–75. 12926995 10.1667/0033-7587(2003)160[0366:rrdmbo]2.0.co;2

[22] Guerrero M, Carlone M. (2010). Mechanistic formulation of a linear-quadratic-linear (LQL) model: split-dose experiments and exponentially decaying sources. Med Phys 37:4173–81. 20879577 10.1118/1.3456927

[23] Brahme A. (2011). Accurate description of the cell survival and biological effect at low and high doses and LET’s. J Radiat Res 52:389–407. 21785229 10.1269/jrr.10129

[24] Garcia LM, Leblanc J, Wilkins D, Raaphorst GP. (2006). Fitting the linear-quadratic model to detailed data sets for different dose ranges. Phys Med Biol 51:2813–23. 16723768 10.1088/0031-9155/51/11/009

[25] Garcia LM, Wilkins DE, Raaphorst GP. (2007). Alpha/beta ratio: a dose range dependence study. Int J Radiat Oncol Biol Phys 67:587–93. 17236975 10.1016/j.ijrobp.2006.10.017

[26] Park C, Papiez L, Zhang S, *et al* (2008). Universal survival curve and single fraction equivalent dose: useful tools in understanding potency of ablative radiotherapy. Int J Radiat Oncol Biol Phys 70:847–52. 18262098 10.1016/j.ijrobp.2007.10.059

[27] Kok HP, Crezee J, Franken NAP, *et al* (2014). Quantifying the com- bined effect of radiation therapy and hyperthermia in terms of equivalent dose distributions. Int J Radiat Oncol Biol Phys 88:739–45. 24411189 10.1016/j.ijrobp.2013.11.212

[28] Crezee J, van Leeuwen CM, Oei AL, *et al* (2016). Biological modelling of the radiation dose escalation effect of regional hyperthermia in cervical cancer. Radiat Oncol 11:14. 26831185 10.1186/s13014-016-0592-z PMC4735973

[29] Dewey WC. (2009). Arrhenius relationships from the molecule and cell to the clinic. Int J Hyperther Mia 25:3–20. 10.1080/02656730902747919 19219695

[30] Henle KJ, Dethlefsen LA. (1980). Time-temperature relationships for heat-induced killing of mammalian cells. Ann N Y Acad Sci 335:234–53. 6931521 10.1111/j.1749-6632.1980.tb50752.x

[31] Sapareto SA, Hopwood LE, Dewey WC, *et al* (1978). Effects of hyperthermia on survival and progression of Chinese hamster ovary cells. Cancer Res 38:393–400. 563767

[32] Qin Z, Balasubramanian SK, Wolkers WF, *et al* (2014). Correlated parameter fit of arrhenius model for thermal denaturation of proteins and cells. Ann Biomed Eng 42:2392–404. 25205396 10.1007/s10439-014-1100-y PMC4709256

[33] He X, Bhowmick S, Bischof JC. (2009). Thermal therapy in urologic systems: a comparison of arrhenius and thermal isoeffective dose models in predicting hyperthermic injury. J Biomech Eng 131:074507. 19640143 10.1115/1.3128671

[34] Mouratidis PXE, Rivens I, ter Haar G. (2015). A study of thermal dose-induced autophagy, apoptosis and necroptosis in colon cancer cells. Int J Hyperthermia 31:476–88. 25974074 10.3109/02656736.2015.1029995

[35] Highfield DP, Holahan EV, Holahan PK, Dewey WC. (1984). Hyperthermic survival of Chinese hamster ovary cells as a function of cellular population density at the time of plating. Radiat Res 97:139–53. 6364200

[36] Borrelli M, Thompson LL, Cain CA, Dewey WC. (1990). Time-temperature analysis of cell killing of BHK cells heated at temperatures in the range of 43C to 57C. Int J Radiat Oncol Biol Phys 19:389–99. 2394618 10.1016/0360-3016(90)90548-x

[37] Dikomey E, Jung H. (1991). Thermal radiosensitization in CHO cells by prior heating at 41–46 degrees C. Int J Radiat Biol 59:815–25. 1672368 10.1080/09553009114550711

[38] Brenner DJ. (2008). The linear-quadratic model is an appropriate methodology for determining isoeffective doses at large doses per fraction. Semin Radiat Oncol 18:234–9. 18725109 10.1016/j.semradonc.2008.04.004 PMC2750078

[39] Kirkpatrick JP, Meyer JJ, Marks LB. (2008). The linear-quadratic model is inappropriate to model high dose per fraction effects in radiosurgery. Semin Radiat Oncol 18:240–3. 18725110 10.1016/j.semradonc.2008.04.005

[40] Xu M, Wright WD, Higashikubo R, Wang L. (1999). Thermal radiosensitization of human tumour cell lines with different sensitivities to 41.1 °C. Int J Hyperthermia 15:279–90. 10458568 10.1080/026567399285657

[41] Xu M, Myerson RJ, Straube WL, *et al* (2002). Radiosensitization of heat resistant human tumour cells by 1 hour at 41.1 degrees C and its effect on DNA repair. Int J Hyperthermia 18:385–403. 12227926 10.1080/02656730210146908

[42] Myerson RJ, Roti JLR, Moros EG, *et al* (2004). Modelling heat-induced radiosensi- tization: clinical implications. Int J Hyperthermia 20:201–12. 15195514 10.1080/02656730310001609353

[43] Franken NAP, Oei AL, Kok HP, *et al* (2013). Cell survival and radiosensitisation: modulation of the linear and quadratic parameters of the LQ model (Review). Int J Oncol 42:1501–15. 23503754 10.3892/ijo.2013.1857

[44] van Leeuwen CM, Crezee J, Oei AL, Franken NAP, *et al* (2017). 3D radiobiolog- ical evaluation of combined radiotherapy and hyperthermia treatments. Int J Hyperthermia 33:160–9. 10.1080/02656736.2016.1241431 27744728

[45] Pajonk F, Ophoven AV, Mcbride WH. (2005). Hyperthermia-induced proteasome inhibition and loss of androgen receptor expression in human prostate cancer cells. Cancer Res 65:4836–44. 15930304 10.1158/0008-5472.CAN-03-2749

[46] Bhuyan BK. (1979). Kinetics of cell kill by hyperthermia. Cancer Res 39:2277–84. 376117

[47] Zhang H, Mehta K, Cohen P, Guha C. (2008). Hyperthermia on immune regulation: a temperature’s story. Cancer Lett 271:191–204. 18597930 10.1016/j.canlet.2008.05.026

[48] Song CW. (1984). Effect of local hyperthermia on blood flow and microenvironment: a review. Cancer Res 44:4721s–30s. 6467226

[49] van den Tempel N, Horsman MR, Kanaar R. (2016). Improving efficacy of hyperthermia in oncology by exploiting biological mechanisms. Int J Hyperthermia 32:446–54. 27086587 10.3109/02656736.2016.1157216

[50] Hildebrandt B, Wust P, Ahlers O, *et al* (2002). The cellular and molecular basis of hyperthermia. Crit Rev Oncol Hematol 43:33–56. 12098606 10.1016/s1040-8428(01)00179-2

